# Breed-dependent associations of production characteristics with on-farm seropositivity for *Ostertagia ostertagi* in dairy cows

**DOI:** 10.1186/s13071-025-06728-9

**Published:** 2025-03-05

**Authors:** Lisa Kuehne, Martina Hoedemaker, Christina Strube, Gabriela Knubben-Schweizer, Andrea Springer, Andreas W. Oehm

**Affiliations:** 1 Clinic for Ruminants with Ambulatory and Herd Health Services, Ludwig-Maximilians-Universität Munich, Oberschleissheim, Germany; 2 Clinic for Cattle, Foundation, University of Veterinary Medicine, Hannover, Germany; 3 Institute for Parasitology, Centre for Infection Medicine, University of Veterinary Medicine Hannover, Hannover, Germany; 4 Institute of Parasitology, Vetsuisse Faculty, University of Zurich, Zurich, Switzerland

**Keywords:** *Ostertagia ostertagi*, Trichostrongyles, Gastrointestinal nematodes, Quantile regression, Production losses

## Abstract

**Background:**

Pasture-borne parasites like *Ostertagia ostertagi* have a negative effect on dairy cow health and productivity. The aim of the present study was to assess potential breed-dependent associations of *O. ostertagi* seropositivity with dairy cow production traits, i.e. milk yield, milk fat and milk protein.

**Methods:**

We describe these associations in German Holstein (GH) cows, a specialised dairy breed, compared with a dual-purpose breed, i.e. German Simmental (SIM). Data from 560 farms across Germany housing 93,030 dairy cows were included. Of the 560 farms, 383 farms housed GH cows and 177 housed SIM. Potential breed-dependent associations of *O. ostertagi* seropositivity with production characteristics were explored via a two-way interaction term using quantile regression. Pasture access, farming type (organic vs. conventional), herd size (number of cows) and study year were included as confounders. The relationship of *O. ostertagi* seropositivity with production traits based on breed was further examined via estimated marginal means.

**Results:**

*Ostertagia ostertagi* bulk tank milk (BTM) seropositivity was associated with lower median milk yield, milk fat and milk protein on GH farms, whereas no differences could be detected between seropositive and seronegative SIM farms. The difference in the production parameters per cow and year at GH farms associated with *O. ostertagi* seropositivity were 631.6 kg milk yield (*P* < 0.001), 20.0 kg milk fat (*P* < 0.001) and 17.0 kg milk protein (*P* = 0.01).

**Conclusions:**

This study indicated differential associations of *O. ostertagi* seropositivity and production level of cows depending on breed. Our results suggest that seropositivity is associated with lower milk yield, milk fat and milk protein in high-performance dairy breeds, whereas no such association may be present in dual-purpose breeds.

**Graphical Abstract:**

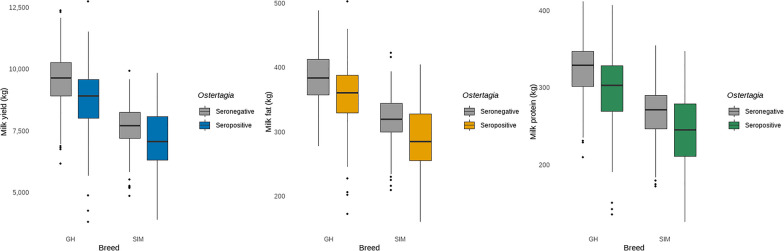

## Background

The growing global emphasis on animal welfare and sustainable food production has prioritised eco-friendly livestock husbandry to conserve natural resources and improve the well-being of farmed animals [1]. Infections caused by helminths exert relevant economic and welfare burdens on the global ruminant livestock sector [1–3]. Gastrointestinal nematodes (GIN) stand out as primary contributors to reduced productivity in ruminants [3–5]. Among these pathogens, *O. ostertagi* is the most prevalent GIN affecting cattle. Studies in The Netherlands and Belgium have determined cow-level prevalences in dairy cows to be as high as 80–100% [6]. Economic losses are mainly indirect because of chronic parasitic gastroenteritis [3, 7, 8]. Only Bellet et al. [7] compared different breeds in association with the economic losses. In this study, dairy cross breeds were more likely to have abomasal lesions due to *Ostertagia* spp. compared with pure dairy breeds, and beef cross breeds were less likely to have *Ostertagia* spp. lesions of higher severity. Villa-Mancera et al. [9] observed the relationship between BTM seropositivity and production losses due to *O. ostertagi* in Mexican cattle. The mean decrease in milk production in the examined herds in Mexico was 0.542 kg/cow/day [9]. In comparison, Charlier et al. [5] described the production loss of the five Flemish provinces (Belgium) in spring and autumn relating to *O. ostertagi* antibodies. The increase of the optical density ratio (ODR) measured by ELISA from 0.702 ODR to 0.958 ODR in spring was associated with a production loss of 1.1 kg/cow/day and the increase from 0.829 ODR to 1.115 ODR in autumn with a loss of 0.9 kg/cow/day.

In this context, it is important to be aware of the widespread presence of anthelmintic resistance in GIN of ruminants [10, 11]. For example, Mauger et al. [11] and Cotter et al. [12] found that anthelmintic resistance against doramectin was present on 91% of the included farms in an Australian study. Furthermore, resistance of *O. ostertagi* against fenbendazole was detected on 80% of farms in Western Australia. Rose Vineer et al. [13] described the anthelmintic resistances of nematodes from cattle, sheep and goats in Europe with a wide range relating to the different anthelmintic classes, i.e. 0–100% against benzimidazoles and macrocyclic lactones except moxidectin, 0–73% against moxidectin and 0–17% against levamisole. Given this scenario and the high prevalence of GIN, alternative ways to combat these infections and to limit their impact on animal health and productivity in farmed ruminant species are necessary [10]. Lins et al. [14] noted differential susceptibility to GIN infection in various sheep breeds. More specifically, Ile de France lambs were compared with Santa Ines lambs regarding their *Haemonchus contortus* infection state. Compared with infected Ile de France lambs, infected Santa Ines lambs had a lower mean number of eggs per gram of faeces and a lower total *H. contortus* worm burden [14]. Previous work indicated potential breed-dependent associations of parasite seropositivity with production parameters in dairy cows [15]. This was examined in greater detail for cattle herds seropositive for *Fasciola hepatica* [16]. Specifically, production decreases associated with *F. hepatica* seropositivity appeared to be more pronounced in German Holstein (GH) cows compared with German Simmental (SIM) cows [16]. While in seropositive GH (compared with seronegative GH) the median reduction per cow and year in milk yield, milk fat and milk protein amounted to 1206.0 kg, 22.9 kg and 41.6 kg, respectively, only milk fat (− 33.8 kg) and milk protein (− 22.6 kg) were affected on seropositive SIM farms compared with seronegative SIM farms. Based on this prior work, the aim of the present study was to evaluate a potential breed-dependent association of *O. ostertagi* seropositivity and dairy cow production traits, i.e. milk yield, milk fat content and milk protein content. We hypothesised that, as shown for *F. hepatica*, seropositivity for *O. ostertagi* would lead to more pronounced production losses in GH cows compared with SIM.

## Methods

### Data selection and extraction

#### Farm systems and data collection

Details about the procedure of sampling and farm selection have previously been specified [16–18]. In brief, 765 farms in three regions of Germany were visited between January 2017 and August 2019. Participation was on a voluntary basis. Study veterinarians visited the different farms and data were collected using paper-based questionnaires and data entry forms. As described by Oehm et al. [18], the characteristics of the farms, e.g. farming type (i.e. conventional versus organic) or the presence of pasture access at the time of the farm visit, were retrieved in a personal conversation with the farm manager. Data were collected in three distinct regions: the North, comprising the states of Schleswig-Holstein and Lower Saxony; the East, including the states of Mecklenburg-Western Pomerania, Brandenburg, Thuringia and Saxony-Anhalt; and the South, representing the federal state of Bavaria. Sample sizes were determined to account for various potential prevalence scenarios (e.g. parasite prevalence) using an 80% statistical power and a 5% significance level. For instance, with an expected prevalence of 30% and a standard deviation of 7, sample size estimations considered precisions of 1, 2, 3 and 4%. Farms were selected to represent a range of herd sizes across regions based on data from the national animal information database (HIT) and regional associations such as the Milchprüfring Bayern e.V. in the south and state control associations in the north and east. To ensure representation across herd sizes, farms were categorized as small, medium or large, based on region-specific herd size cutoffs derived from HIT data. These cutoffs divided the target population into three equal groups by herd size:

North: small (1–64 cows), medium (65–113 cows), large (≥ 114 cows);

East: small (1–160 cows), medium (161–373 cows), large (≥ 374 cows);

South: small (1–29 cows), medium (30–52 cows), large (≥ 53 cows).

To account for an anticipated response rate of 20–40%, a random sample of 1250 farms per region (five times the required number) was drawn, with the final sample size set at 250 farms per region. An automated randomisation algorithm ensured unbiased selection. This strategy provided a diverse representation of herd sizes and facilitated feasibility regarding logistics, including the number of farms visited per day during the 3-year study period.

After the visit, questionnaires and data entry forms were manually inserted into a central database. Production data such as milk yield (in kg), milk fat (in kg) and milk protein (in kg) were accessed from the national milk recording system for up to 3 years prior to the farm visit date (Dairy Herd Improvement, DHI). The national cattle registration database (HI-Tier) provided information about the breed on an individual cow level.

#### Serology for *Ostertagia ostertagi*

Bulk tank milk (BTM) samples were taken once by the farm manager at the end of the grazing season from August to November in the year of the farm visit. During this period, antibody titres of *O. ostertagi* are at the highest level [19]. Collection of the samples, arrival at the laboratory, treatment of the samples and the analysis have previously been described [15]. Antibodies against *O. ostertagi* were measured using a commercial enzyme-linked immunosorbent assay (ELISA) kit based on crude adult worm extract according to the manufacturer's instructions (SVANOVIR^®^ *O. ostertagi*-Ab, Boehringer Ingelheim Svanova, Uppsala, Sweden). An ODR ≥ 0.5 identifies herds as likely to experience a negative effect on herd milk yield [20, 21].

### Data management

Plausibility of the collected data was established on different levels as described in a prior study [16]. Implausibilities or missing values led to an exclusion of the corresponding observations from further processing. Information on milk yield, milk fat and milk protein (each in kg/cow/year) content was available at the farm level (adjusted for the number of cows per farm, hence representing the individual cow level as well) for up to 3 years prior to the farm visit. A simple median for each farm for these three values was created for further analyses. Farms were categorised into GH or SIM if at least 85% of the cows were of either breed on the day of the farm visit. Based on the ODR threshold of ≥ 0.5, a binomial variable (*O. ostertagi* seropositivity/negativity) was generated.

### Statistical analysis

Target variables [milk yield (in kg), milk fat (in kg), milk protein (in kg)] were modelled using a quantile regression approach where the median quantile of the dependent variable is modelled given the predictors [22, 23]. Target variables were calculated from 3 years of production data prior to the year of the farm visit. This approach aimed to reduce variability caused by transient annual factors and to reflect a typical level of farm production. Median regression was chosen because of the nature of our data and the objectives of the study as it was particularly well suited to our cross-sectional design, providing robust estimates that were less sensitive to the presence of extreme values, ensuring reliable inference from the data. Median regression, unlike ordinary least squares regression, focusses on the conditional median of the target variable rather than the mean, making it robust to outliers and skewed distributions—characteristics often observed in farm-level production data [22, 24, 25]. To examine a potential breed-dependent effect on production traits, a two-way interaction term (Breed*O. ostertagi seropositivity) was incorporated. Potential confounders at farm level included the presence of pasture access on the farm (present vs. absent), farming type (organic vs. conventional), herd size (number of cows) and visit year. The inclusion of the interaction term was central to the model's ability to identify whether the relationship between *O. ostertagi* seropositivity and production outcomes varied across breeds. Additionally, adjusting for confounders like herd size and farming type ensured that observed associations were not spuriously influenced by these factors. Year of sampling was included to account for temporal trends or conditions that might affect both serostatus and production. Confounders entered the model in a backwards selection fashion. One confounder at a time was removed from the model, and the Akaike´s information criterion (AIC) and the Bayesian information criterion (BIC) were used to compare and select models. Candidate models were ranked using the compare_performance() function from the R package performance [26]. To examine how the relationship of *O. ostertagi* seropositivity depended on the two breeds (GH and SIM), we applied the emmeans() function from the emmeans package [27] to further explore the nature of the interaction. The problem of multiple comparisons was managed using the Benjamini-Hochberg method to correct *P*-values [28]. We also tested the interaction between farming type and pasture access in relation to the target variables to explore whether their effects on production outcomes varied depending on the combination of these factors. However, the interaction term did not translate into superior models based on AIC and BIC. Therefore, this interaction was excluded from the final model to maintain a more parsimonious model focussing on the main effects of farm type and pasture access, along with other relevant confounders, ensuring both interpretability and robustness of the results.

All the analyses and the visualisation were executed in R Software for Statistical Computing version 4.3.3 and the R Studio interface [29, 30]. Throughout the analyses, statistical significance was set at *P* ≤ 0.05.

## Results

### Descriptive results

Descriptive statistics of the data set have previously been reported [15–18]. In brief, 765 farms were visited and BTM data were available from 645. Of these, 49 farms did not appear in the national milk recording system. Furthermore, there were 36 mixed-breed farms. In the present work, 560 farms with a total of 93,030 dairy cows are represented. Most of the farms were assigned to the breed GH (383 farms, 68.3%) with 177 operations (31.6%) housing SIM. Mean herd size was 167 cows with a minimum of five and a maximum of 2821 animals. The main housing system was free stall facilities (447 farms; 79.8%), followed by other farming types like pasture-based systems (60 farms; 10.7%) and tie stall barns (54 farms; 9.6%). Two hundred ninety-five of the 560 farms (52.7%) offered access to pasture, and 42 farms (7.5%) pursued organic farming principles. The presence of *O. ostertagi* antibody levels ≥ 0.5 ODR was confirmed on 211 farms (38.5%). A descriptive overview of the continuous variables is shown in Table 1.

**Table 1:** Descriptive overview of continuous variables in the data set (n_farms_ = 560)

Variable	Mean ± SD	Median	IQR	Min–max
Milk yield^1,2^	8684.0 ± 1444.0	8763.0	2007.0	3940.0–12,527.0
Milk fat^1,2^	352.4 ± 54.0	356.4	70.2	161.0–490.3
Milk protein^1,2^	296.9 ± 48.5	301.7	64.5	128.4–412.3
Herd size^3^	167.3 ± 252.0	81.0	142.0	5.0–2821.0

^1^Median value per farm

^2^In kg

^3^Number of lactating and dry cows

### Association of *Ostertagia ostertagi* seropositivity with production parameters

Model results for the milk yield are shown in Table 2. The relevant confounders of the milk yield model were farming type, herd size and year. The organic farming type was associated with a lower milk yield of − 1284.5 kg/cow/year compared with the conventional farming type (*P* = 0.002; 95% confidence interval [CI] − 2096.28– − 472.65). Larger herd size was associated with a higher milk yield (0.29 kg/cow/year; *P* < 0.001; CI 0.19–0.39). Furthermore, study year 3 was associated with lower milk yield (− 348.96 kg/cow/year; *P* = 0.037; CI − 675.90– − 22.02) compared with year 1. The interaction between *O. ostertagi* seropositivity and the SIM breed was associated with a higher milk yield compared to the breed GH (584.19 kg/cow/year; *P* = 0.048; CI 6.27–1162.11).

**Table 2:** Model results of the relationship between *Ostertagia ostertagi* seropositivity, breed and potential confounders with median milk yield per cow per year

Variable	Category	Estimate	95% CI^a^	*P*-value
Intercept		9625.62	9408.85–9842.39	< 0.001*
*Ostertagia* seropositivity/-negativity	Seronegativity	Reference	–	–
	Seropositivity	− 631.56	− 929.82–− 333.31	< 0.001*
Breed	GH	Reference	–	–
	SIM	− 1841.28	− 2130.35–− 1552.20	< 0.001*
Farming type	Conventional	Reference	–	–
	Organic	− 1284.46	-2096.28 – –472.65	0.002*
Herd size^b^	Continuous	0.29	0.19–0.39	< 0.001*
Study year	1	Reference	–	–
	2	0.90	− 246.09–247.89	0.994
	3	− 348.96	− 675.90–− 22.02	0.04*
Interaction	*Ostertagia ostertagi* seropositive x SIM	584.19	6.27–1162.11	0.05*

Milk yield (in kg per cow per year)

*GH* German Holstein, *SIM* German Simmental

^a^*CI* Confidence Interval

^b^Number of cows

^*^Statistically significant

**Fig. 1:**
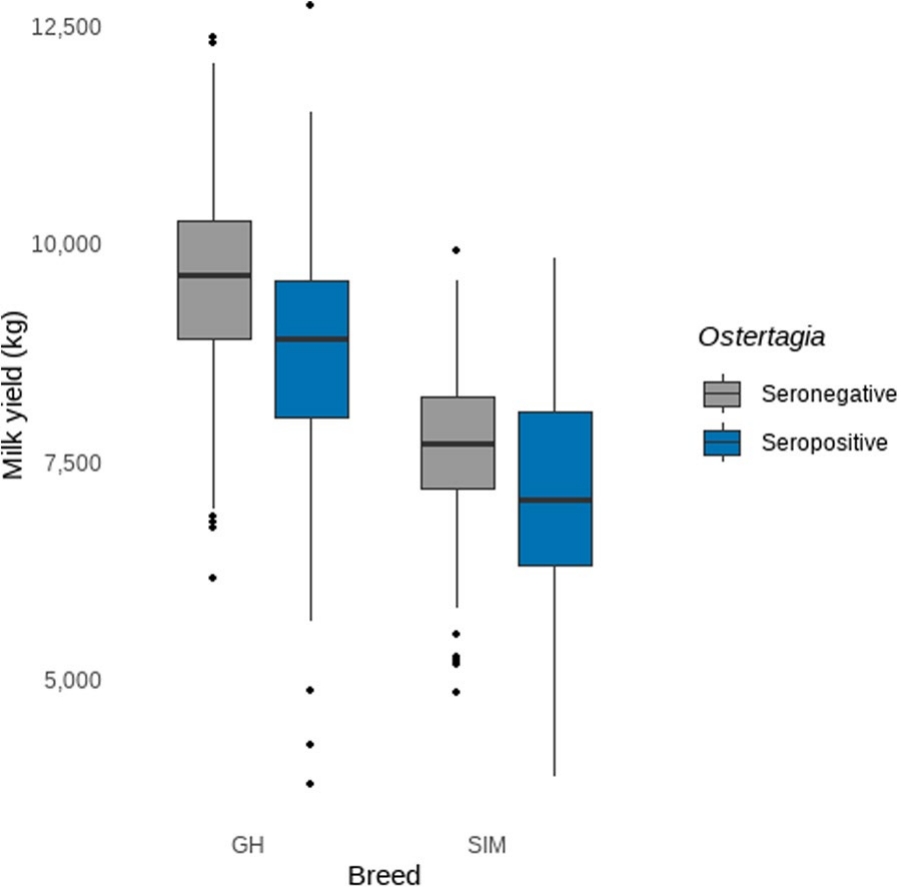
Exploration of the interaction term of *Ostertagia ostertagi* seropositivity/seronegativity with breed in the milk yield model (in kg per cow per year). *GH* German Holstein, *SIM* German Simmental

The exploration of the interaction term between breed and *O. ostertagi* seropositivity/-negativity regarding milk yield is shown in Fig. 1. On GH farms, *O. ostertagi* seropositivity was associated with a lower median milk yield of − 631.6 kg/cow/year (*P* = 0.0002; CI 239–1024; standard error [SE] = 152) compared with seronegative farms. Seropositive GH farms showed a median milk yield of 8871 kg/cow/year (CI 8639–9103; SE = 118), whereas seronegative GH farms produced a median milk yield of 9502 kg/cow/year (CI 9302–9703; SE = 102). SIM farms had a generally lower milk production than GH farms. *Ostertagia ostertagi* seropositivity was not associated with median milk production of SIM (*P* = 0.9977; CI – 610–705; SE = 255) compared with seronegative SIM operations. Seronegative SIM farms produced 7661 kg/cow/year of milk (CI 7442–7880; SE = 112), which amounted to a difference of 1841.3 kg/cow/year (*P* < 0.0001; CI 1461–2221; SE = 147) milk between seronegative GH and SIM operations. Seropositive SIM farms produced a median milk yield of 7614 kg/cow/year (CI 7170–8057; SE = 226). This represents a total median difference of 1257.1 kg/cow/year (*P* < 0.0001; CI 592–1922; SE = 258) milk yield between GH and SIM when both breeds were seropositive.

The milk fat model incorporated one single confounder, i.e. farming type, shown in Table 3. Organic farming was associated with lower milk fat (− 60.00 kg/cow/year; *P* < 0.001; CI − 87.48–− 32.52) compared with conventional farming.

**Table 3:** Model results of the relationship between *Ostertagia ostertagi* seropositivity, breed and potential confounders with median milk fat per cow per year

Predictor	Category	Estimate	95% CI^a^	*P*-value
Intercept		384.00	377.96–390.04	< 0.001*
*Ostertagia* seropositivity/-negativity	Seronegativity	Reference	–	–
	Seropositivity	− 20.00	− 29.58––10.42	< 0.001*
Breed	GH	Reference	–-	–
	SIM	− 62.00	− 69.94–− 54.06	< 0.001*
Farming type	Conventional	Reference	–	–
	Organic	− 60.00	− 87.48–− 32.52	< 0.001*
Interaction	*Ostertagia ostertagi* seropositive x SIM	10.00	− 9.88–29.88	0.325

Milk fat (in kg per cow per year)

*GH* German Holstein, *SIM* German Simmental

^a^*CI* Confidence Interval

^*^Statistically significant

**Fig. 2:**
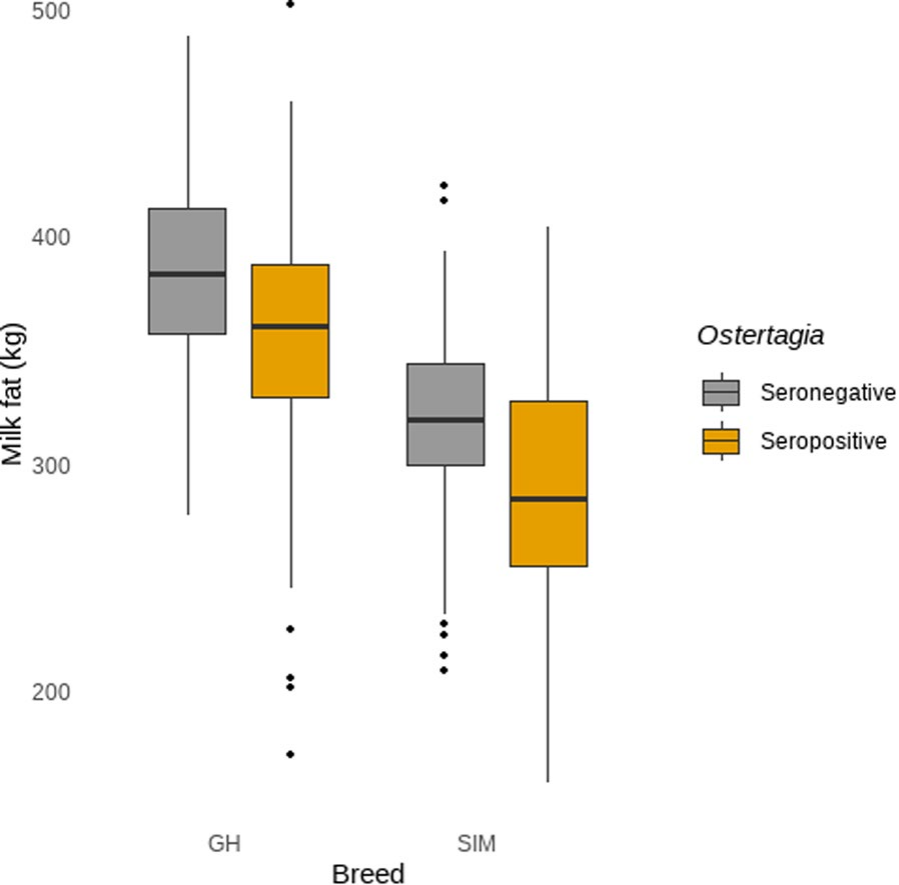
Exploration of the interaction term of *Ostertagia ostertagi* seropositivity/seronegativity with breed in the milk fat model (in kg per cow per year). *GH* German Holstein, *SIM* German Simmental

The exploration of the interaction of *O. ostertagi* seropositivity/-negativity and breed regarding milk fat is shown in Fig. 2. More specifically, *O. ostertagi* seropositivity was associated with lower median milk fat on GH farms (− 20.0 kg/cow/year *P* < 0.001; CI 7.4–32.6; SE = 4.89). This means that seropositive GH farms had a median milk fat of 360 kg/cow/year (CI 352–367; SE = 3.78) compared with 380 kg/cow/year (CI 373–386; SE = 3.25) in seronegative farms. Such an association was not evident on SIM farms. (*P* = 0.77). In comparison, the median milk fat difference of seronegative GH and SIM amounted 62 kg/cow/year (*P* < 0.0001; CI 51.6–72.4; SE = 4.05). The median milk fat difference of seropositive GH and SIM counted 52 kg/cow/year (*P* < 0.0001; CI 28–76; SE = 9.31), with a higher milk fat production of GH in contrast to SIM.

The confounders of the milk protein model were farming type and pasture access. The model results are shown in Table 4. Organic management was associated with lower milk protein compared with conventional management (− 57.00 kg/cow/year; *P* = 0.001; CI − 89.08–− 24.92). Access to pasture was associated with lower milk protein (− 10.00 kg/cow/year; *P* = 0.027; CI − 18.82– − 1.18). The interaction between *O. ostertagi* seropositivity and SIM tended to be associated with higher milk protein compared to the GH farms (18.00 kg/cow/year; *P* = 0.084; CI − 2.35–38.35).

**Table 4:** Model results of the relationship between *Ostertagia ostertagi* seropositivity, breed and potential confounders with median milk protein per cow per year

Predictor	Category	Estimate	95% CI^a^	*P*-value
Intercept		334.00	328.75–339.25	< 0.001*
*Ostertagia* seropositivity/-negativity	Seronegativity	Reference	–	–
	Seropositivity	− 17.00	− 27.78–− 6.22	0.002*
Breed	GH	Reference	–	–
	SIM	− 60.00	− 69.26–− 50.74	< 0.001*
Farming type	Conventional	Reference	–	–
	Organic	− 57.00	− 89.08–− 24.92	0.001*
Pasture access	Absent	Reference	–	–
	Present	− 10.00	− 18.82–− 1.18	0.03*
Interaction	*Ostertagia ostertagi* seropositive x SIM	18.00	− 2.35–38.35	0.084

Milk protein (in kg per cow per year)

*GH* German Holstein, *SIM* German Simmental

^a^*CI* Confidence Interval

^*^Statistically significant

**Fig. 3:**
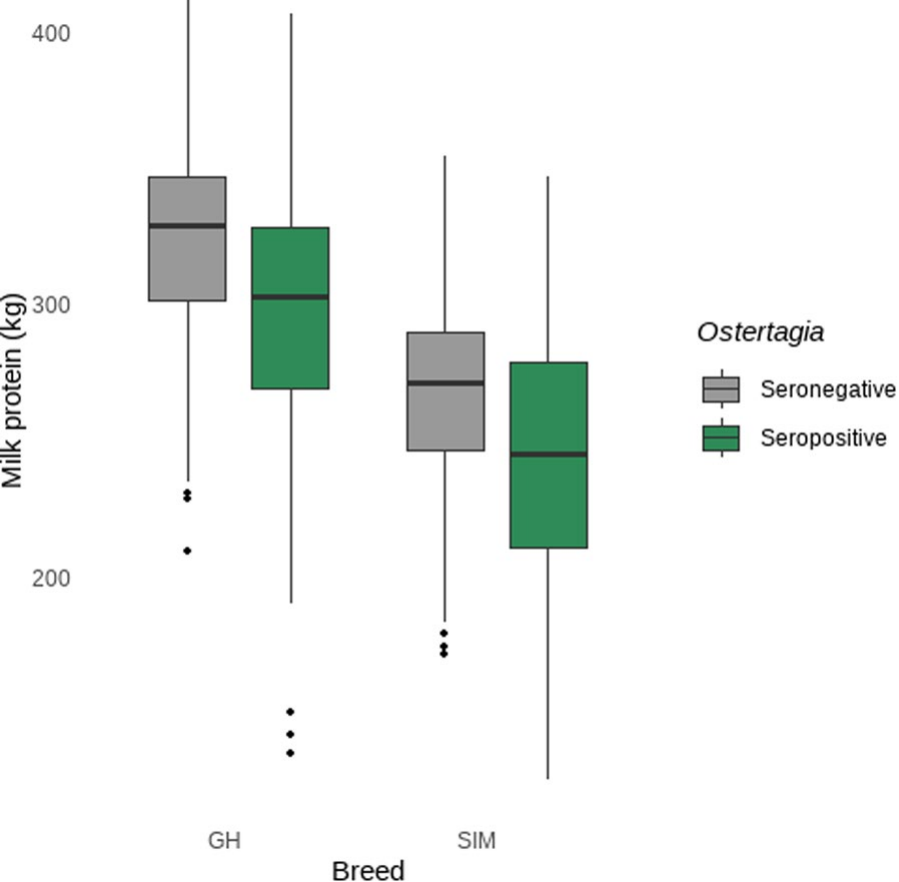
Exploration of the interaction term of *Ostertagia ostertagi* seropositivity/seronegativity with breed in the milk protein model (in kg per cow per year). *GH* German Holstein, *SIM* German Simmental

The interaction of *O. ostertagi* seropositivity/negativity and breed regarding milk protein is illustrated in Fig. 3. Milk protein of *O. ostertagi*-seropositive GH farms was 308 kg milk protein per cow per year (CI 299–317; SE = 4.61) compared with 325 kg/cow/year on seronegative GH farms (CI 319–330; SE = 2.94), i.e. a median difference of 17 kg/cow/year milk protein on GH farms (*P* = 0.0113; CI 2.82–31.2; SE = 5.50). In comparison, SIM farms showed no difference between seropositive and seronegative operations (*P* = 0.9996). The difference of seronegative GH farms versus seronegative SIM farms in relation to the milk protein comprised 60 kg/cow/year (*P* < 0.0001; CI 47.82–72.2; SE = 4.72) milk protein. This means seronegative GH farms produced a median of 60 kg more milk protein than seronegative SIM. The difference between seropositive GH and seropositive SIM amounted to 42 kg/cow/year milk protein (*P* < 0.0001; CI 17.94–66.1; SE = 9.34), with the GH farms showing more production of milk protein. The milk protein amounts of *O. ostertagi* seronegative SIM farms were 265 kg/cow/year (CI 255–274; SE = 4.62) compared with seropositive SIM farms with 266 kg/cow/year (CI 250–281; SE = 7.94) milk protein.

## Discussion

The aim of this study was to examine potential breed-dependent associations of *O. ostertagi* seropositivity with production traits in dairy cows. The breeds compared in this study were GH and SIM.

As hypothesised and similar to *F. hepatica* [16], we detected associations between seropositivity for *O. ostertagi* and lower production traits in GH cows compared with SIM. More specifically, associations between *O. ostertagi* seropositivity and production level were absent in SIM cattle, underscoring a notable breed-dependent link. BTM seropositivity for *O. ostertagi* was associated with a lower median milk yield, milk fat and milk protein in GH cows compared with animals in seronegative herds. These results suggest that GH cows may be more susceptible to productivity losses associated with iGIN infections, potentially due to breed-specific differences in metabolism, immune response, or production demands. GH cows have been predominantly selected for high production output with milk yield as well as milk components, i.e. milk fat and milk protein, in the focus of genetic selection [31–33]. However, several studies have provided evidence that the general fitness of GH cows is lower compared with other less specialised breeds [34–36]. Manuelian et al. [37] found that specialised dairy cows produced milk with higher fat and protein content compared with dual-purpose breeds, which may reflect differences in their metabolic pathways and nutritional requirements. This higher production capacity places additional demands on their metabolism, potentially leading to increased susceptibility to metabolic disorders and health issues if not managed properly. Furthermore, considerable variation in immune response traits have been suggested among dairy cattle, with specialised dairy breeds exhibiting a higher incidence of certain disease because of their intense production demands. This may be attributed to the trade-off between energy allocation for milk production and immune function, where high-producing cows may divert energy away from immune responses, making them more vulnerable to infections. SIM cows on the other hand are characterised by a more balanced level of milk and meat production. The body condition of this dual-purpose breed is higher than that of breeds selected for the highest milk yield [38]. Eevidence suggests that SIMs are better able to cope with periods of negative energy balance compared with specialised high-yielding dairy cows (relating to mastitis, endometritis and ketosis) and that they appear to mobilise fewer body reserves during the lactation period [39–41]. Given the results from our study and considering that, unlike GH cows, SIM cows as a dual-purpose breed did not show considerable links between *O. ostertagi* seropositivity and production traits, we hypothesise that dual purpose breeds may be more resilient to infections with GIN.

This pattern indicates the potential relevance of considering breed-specific management and prevention strategies in parasitic control programmes, especially for high-yielding dairy breeds like GH, which appear to be more affected by infections in terms of production losses. While further research is necessary to elucidate the underlying mechanisms, the present work indicates that breed-specific traits are of interest in the context of mediating the productivity impact of GIN infections in dairy cattle. Given that to our knowledge this study is among the very first of its kind, it opens directions for further research into the underlying epidemiological, genetic and immunological factors that may contribute to breed-based differences in parasitic tolerance. Over the long term, understanding these mechanisms may translate into the development of selective breeding programmes aimed at enhancing parasite resistance in vulnerable breeds like GH, thereby improving overall productivity and animal health. Additionally, sustainable parasitic control practices that are tailored to specific breeds can be designed, potentially minimising economic losses associated with parasitism in dairy production. For example, monitoring strategies may be adjusted depending on breed with specialised breeds being more closely or intensely monitored regarding GIN. This may include strategic anthelmintic treatments based on diagnostic indicators (e.g. faecal egg counts). Moreover, rotational grazing or co-grazing with other species may lower the infection pressure and reduce the potential impact of GIN. This may be complemented with optimised feeding strategies for specialised dairy cows supporting the nutritional and metabolic demands.

Further studies may also explore whether *O. ostertagi* is associated with the same production losses in breeds other than GH and SIM and whether similar patterns hold true for other gastrointestinal parasites and in different environmental or management contexts, which would enhance the generalisability of these insights and solidify the role of breed-specific considerations in livestock parasitology and management.

Besides *O. ostertagi* seropositivity/negativity, organic farming was associated with lower milk yield, milk fat and milk protein. Our results are in accordance with previous research, while it is worth mentioning that the existing body of literature on breed-specific differences is very scarce. Previous work reported that organic farms have a lower level of milk production compared with conventionally run operations [42–45]. One presumed reason for the lower milk yield in organic farms is the use of organic feed, which translates into lower energy components compared with the highly concentrated supplemented feed used in conventional farming. Conventional farms commonly provide more grain and maize silage and often include professional nutritionists to establish an efficient ration and for feeding advice [42–44]. There are various opinions about the difference between organic and conventional farming regarding milk fat [46]. Lock et al. [47] suggest an association between highly concentrated fat supplemented feed at some conventional farms with higher milk fat compared with organic farms. On the other hand, some studies have indicated an increase in milk fat in organic farming systems, attributed to the use of breeds other than GH [48–50].

Similarly, different levels of milk protein have been observed between conventional and organic farms. Schwendel et al. [46] reviewed various possibilities for the relationship between milk protein and farming systems or feeding management. On the one hand, most of the reviewed studies described higher milk protein levels in conventional farming systems [51, 52] and our results are in accordance with these. On the other hand, only Vicini et al. [53] showed a higher protein concentration in organic than in conventional milk. Furthermore, Walker et al. [54] found no association between the protein concentration and composition in the milk and feeding management. For milk protein, not only the feeding management is a limiting factor but also the genetic variation within and between breeds, suggested by a study identifying variants of the caseins in milk of 144 Norwegian cows [55]. Our study aligns with these results.

Pasture access was associated with a lower median milk protein content. Different opinions exist about the influence of grazing or non-grazing on milk protein [46]. On the one hand, non-grazing systems have been discussed to feed more concentrated and supplemented feed [42]. Furthermore, organic farming principles, which commonly incorporate pasturing of cattle, have previously been associated with a decrease of milk protein compared with conventional and non-grazing farming procedures, respectively [51, 52]. However, Walker et al. [54] could not confirm an effect of nutrition and management on the amount of protein in the milk but on milk yield. To improve the estimation of the nutritional effects on milk protein content, future analyses could benefit from incorporating more detailed parameters such as the amount of pasture consumed, total feed intake and the time spent on pasture. Additionally, information on the supplementation of energy feeds like concentrates or silage would provide valuable insights into the cows’ overall nutritional status. However, collecting these data was beyond the scope of the current study. Including such factors in future research would allow for a more precise understanding of the role of different feeding regimes in milk protein production and enhance the accuracy of nutritional effect estimates.

A larger herd size was linked to higher median milk yield. Some studies have explored the relationship between herd size and milk production aiming to determine the impact of herd size on milk yield [56, 57]. The relationship between herd size and milk yield may well be mediated by several other factors like improved housing and management or a more industrialised, output-oriented way of dairy farming acting as a proxy for the covariate herd size.

Study year 3 was associated with a lower median milk yield compared with study year 1, likely acting as a proxy for various environmental, economic and management-related factors. For instance, weather variations such as the extreme heat and dryness observed in study year 3 [58] may have directly impacted milk production through heat stress in cows [59–61] or indirectly through reduced feed quality and availability. Additionally, external factors such as increased disease burdens or fluctuations in milk prices might have influenced overall productivity. These between-year variations underscore the complexity of the production system and their potential influence on our model estimates. While including study year as a confounder aimed to account for such year-specific effects, these variations may still affect the extrapolability of our findings to other contexts. Furthermore, the hot and dry conditions in study year 3 could have impacted the survival and infectiousness of *O. ostertagi* larvae, possibly altering exposure risks and, consequently, the observed associations between seropositivity and production outcomes. However, the weather-related effects on these dynamics were beyond the scope of this study and the impact of such variations on infection dynamics and impact on productivity should be investigated in the context of further investigations integrating longitudinal weather data, pasture contamination levels and parasite burden to provide insights into these interrelated dynamics.

When interpreting the results from this study, some aspects need to be taken into consideration. The cross-sectional design of our study involved collecting bulk tank milk (BTM) samples for *O. ostertagi* seropositivity/negativity and confounder data during the year of the farm visit, while milk production data (milk yield, milk fat and milk protein) spanned the 3 years preceding the visit. To ensure a stable and representative measure of farm-level production, we calculated the median production values over this 3-year period. This approach reduced the influence of short-term variability caused by factors such as weather, feed changes or transient management practices, allowing us to capture the typical performance of each farm. Although this design may introduce a temporal discrepancy between the single timepoint BTM seropositivity/negativity and the multi-year production data, the choice is epidemiologically justified. *Ostertagia ostertagi* seropositivity/negativity is likely reflective of a longitudinal condition rather than a transient state, as farms generally maintain consistent seropositive or seronegative statuses due to chronic or repeated exposure within herds [62]. This longitudinal nature is also true for breed as well as confounding variables such as herd size, farming type and the presence of pasture access on farm. It is important to be aware that using production data spanning multiple years may introduce some temporal complexity. However, to account for this, we included visit year as a confounder in our models, mitigating potential biases associated with temporal mismatches. Restricting the analysis to a single year might have aligned the data more tightly but would have amplified the effects of random annual fluctuations, potentially obscuring the underlying patterns of association we sought to investigate. By incorporating multi-year production data and controlling for confounders such as herd size, farming type and pasture access, our approach allowed for robust evaluation of potential breed-dependent associations between *O. ostertagi* exposure and production outcomes while reflecting the broader and ongoing conditions on farms. Moreover, in the context of cross-sectional studies, it is crucial to understand that solely associations between variables rather than causalities can be inferred from the modelling results. To investigate the potential causal nature of associations, specific study designs are necessary.

Seropositivity in the present study was defined as BTM seropositivity of a farm for *O. ostertagi* using a commercially available ELISA [20, 62]. ELISA-based determination of seropositivity using BTM samples has been a common procedure in parasitological research [63, 64]. The benefits and disadvantages of ELISA versus other methods like faecal egg count or pepsinogen levels have been discussed in previous studies [65–67]. As Charlier et al. [63] elaborated, the ELISA method has good repeatability over replicates, plates and days with BTM samples. There might, however, be cross reactions with other helminthic infections, i.e. *Cooperia* species and *F. hepatica* [68]. Likewise, Bennema et al. [69] reported that the ELISA may not always be able to clearly differentiate between parasites, especially GIN. Complementing our analyses with animal-level coproscopic examinations could have benefitted this work. However, as the underlying study did not primarily focus on parasitological questions, this was beyond the scope of data collection in the context of this work.

While a binary classification of a farm as either seropositive or seronegative for *O. ostertagi* is commonly used in parasitological studies, it has limitations compared with a quantitative ELISA result. The binary classification does not provide information on the intensity of infection, which could vary within a herd and affect the severity of the associated productivity impacts. Quantitative information could offer more precise data on the level of exposure or infection, potentially providing a finer understanding of the relationship between *O. ostertagi* seropositivity and milk production outcomes. Using a binary classification, therefore, might result in a loss of sensitivity in detecting subtle variations in the magnitude of the observed effects on productivity. Future studies could benefit from integrating both qualitative and quantitative data to more accurately capture the dynamics of parasitic infection and its impact on farm productivity.

Furthermore, it would be interesting to comparatively assess herd-level BTM seropositivity/negativity and parasitological results of each single cow. From the present results, it appears that SIM cows experience less pronounced impacts on production associated with *O. ostertagi* seropositivity in contrast to GH cows. Since this work is one of the first of its type, future efforts should concentrate more deeply on the underlying mechanisms of the results obtained in the present work.

## Conclusions

In this study, we demonstrate breed-dependent associations between *O. ostertagi* seropositivity and milk production traits in dairy cows. We showed that *O. ostertagi* seropositivity was associated with more pronounced production losses in GH, a high-yield breed, than in SIM, a dual-purposed breed. The reason for this difference between the two breeds may be that the GH cows are more susceptible to parasitic burdens because of their genetic selection, whereas SIM cows may be more resilient.

## Data Availability

The data were collected from individual dairy farms, with participants providing written consent for the use of farm-specific data under the condition that it would not be shared with third parties. Consequently, data cannot be transferred to others without a formal agreement. Qualified researchers interested in accessing the data must sign a contract with the University of Veterinary Medicine Hannover, which ensures confidentiality in compliance with German data protection laws. Currently, no data access committee is in place, but one will be established for this purpose. The committee will include the authors, members from the University of Veterinary Medicine Hannover and representatives from the funding institution. Interested parties who are able to enter into such a contract can contact LK, lothar.kreienbrock@tiho-hannover.de.

## Abbreviations

BTM: Bulk Tank Milk
DHI: Dairy Herd Improvement
GH: German Holstein
GIN: Gastrointestinal Nematodes
ODR: Optical Density Ratio
SIM: German Simmental
